# How important is school environment in explaining individual variance of health behaviors?

**DOI:** 10.11606/S1518-8787.2019053001568

**Published:** 2019-11-18

**Authors:** Antonio Fernando Boing, SV Subramanian, Alexandra Crispim Boing

**Affiliations:** I Harvard University. T.H. Chan Harvard School of Public Health. Department of Social and Behavioral Sciences. Boston, MA, United States; II Universidade Federal de Santa Catarina. Programa de Pós-graduação em Saúde Pública. Departamento de Saúde Pública. Florianópolis, SC, Brasil.

**Keywords:** Adolescent health, Health behaviors, Schools, Epidemiology, Multilevel analysis

## Abstract

We analyzed data from the National School-based Health Survey (PeNSE) carried out in Brazil in 2015 (n = 102,072 adolescents) to estimate how much of the individual variance in the prevalence of health behaviors is attributable to the school level. Multilevel logistic regression models were calculated to estimate the variance partitional coefficient (VPC) of the use of drugs, intake of unhealthy food, leisure physical activity and weight-related behaviors. The between-schools variance was significant in all tested models. The highest VPCs were observed when the use of drugs was analyzed (15%-20% of the total variance of smoking and use of illegal drugs). Lower, but still significant, values were observed in the other outcomes. The school context plays an important role in the adolescents’ health and should be considered in the design of public policies and actions in public health.

## INTRODUCTION

Adolescence is a critical period of life in which schools and peers come to play a central role in decision making. Researches have shown that the school environment may have profound impact on adolescents’ health behaviors^1^ . This is particularly important because many health behaviors adopted at this point in life impact the whole life of the individual having devastating effects on health.

Each school has specific set of values, curriculum, policies, norms, neighborhood, staff and student composition that altogether influence health outcomes^1^ . However, when analyzing health behaviors among adolescents, few studies have explored and reported how much of the individual variance is attributable to the school level. Furthermore, when analyzing adolescent health, the evidence from studies carried out in low-and middle-income countries (LMIC) are particularly limited^2^ . The main purpose of this study is to fill this gap in research by using multilevel models to partition the variance of health behaviors among adolescents in a LMIC.

## METHODS

We used data from the National School-based Health Survey (PeNSE) carried out in 2015 in a nationally representative sample of Brazilian students enrolled in the 9th grade of elementary schools. The sample included public and private schools. Evening classrooms and schools with less than 15 students in the 9^th^ grade (around 3% of the total students) were excluded. The Federal District and the 26 State capitals formed 27 geographic strata. The other cities were grouped into 26 additional strata, each one representing one Brazilian state. According to the school’s administration type (public or private) and quantity of classes, a two-stage cluster sample was selected in each one of the geographic strata. Schools were the primary sample unit and classes the second stage. All students in the selected classrooms were invited to participate in the study. The adolescents answered the questionnaire autonomously via an electronic device.

The outcomes were the (i) use of legal and illegal drugs in the last 30 days (smoking, alcohol and illegal drugs), (ii) regular intake of unhealthy food (≥ 5 days/week of deep-fried salty snacks, ultraprocessed foods, candies or soft drink), (iii) regular intake of healthy food (≥ 5 days/week of fruits or vegetables), (iv) leisure physical activity (≥ 300min/week) and (v) weight-related behaviors in the last 30 days (use of laxative to lose weight, use of medicine to gain or lose weight).

We performed multilevel logistic regression analyzes to calculate the variance partitional coefficient (VPC), considering the adolescents as the first level of analysis and the schools as the second level. The VPC informed us on the proportion of total variance in each one of the outcomes that is attributable to the school level. The analyses were stratified by type of school (public, private) and adjusted by mother’s educational level, gender, age and skin color/race. We used multiple imputation to account for missing data observed in the variable ‘mother’s level of education’. When performing the imputation, we used household goods and services (housemaid at least three times per week, internet connection, car, home telephone, mobile phone and number of bathrooms with a shower) as predictive variables.

The National School-based Health Survey (PeNSE) 2015 was approved by the National Research Ethics Committee (protocol 1.006.467).

## RESULTS

Data from 102,072 students and 3,040 schools were analyzed. Most of the adolescents were girls (51.3%), aged between 14 and 16 years (78.0%), studied in public schools (74.2%) and were dark or light skinned black (56.4%). Regarding the schools, most of them were public (74.4%), had more than 500 students (60.4%) and were located in the urban region (86.4%).

The prevalence of smoking, alcohol consumption and the use of illegal drugs was 5.6%, 23.8% and 4.2%, respectively ( Table ). All the three behaviors were more common among students of public schools. The consumption of unhealthy foods ranged from 13.7% (deep-fried salty snacks) to 41.6% (candies). Around 1/3 of the students eat fruits or vegetables at least five days a week and one out of six are physically active. The use of laxative to lose weight was reported by 7.0% of the sample (higher among public school students), whereas 6.0% reported use of medicines to lose weight.

**Table:** Prevalence and variance partition coefficients estimates from multilevel models of health risk behaviors. Brazil, 2015

	All	Public school			Private school		
		
	Prevalence	Prevalence	Empty model	Adjusted model*	Prevalence	Empty model	Adjusted model*
	% (IC_95%_)	% (IC_95%_)	VPC (IC_95%_)	VPC (IC_95%_)	% (IC_95%_)	VPC (IC_95%_)	VPC (IC_95%_)
*Use of legal and illegal drugs in the last 30 days*							
Smoking	5.6 (5.2–6.0)	5.9 (5.7-6.3)	11.3	10.9	3.6 (3.0-4.4)	19.0	17.2
Alcoholic beverages	23.8 (23.2-24.4)	24.3 (23.6-24.9)	6.3	5.9	21.2 (19.6-22.9)	8.6	8.5
Illegal drug	4.2 (3.9-4.4)	4.3 (4.0-4.6)	15.4	15.3	3.4 (2.9-3.9)	21.2	20.6
*Regular intake of unhealthy food (>= 5 days per week)*							
Deep-fried salty snacks	13.7 (13.2-14.2)	13.7 (13.2-14.2)	3.6	3.7	13.8 (12.7-15.0	8.5	8.8
Ultraprocessed foods	31.3 (30.7-32.0)	29.8 (29.2-30.5)	3.7	3.2	39.9 (38.4-41.5)	3.1	3.1
Candies	41.6 (41.0-42.3)	41.6 (40.9-42.3)	3.0	2.9	41.6 (39.9-43.4)	2.2	2.3
Soft drink	26.7 (26.0-27.3)	26.5 (25.8-27.2)	5.4	5.1	27.5 (25.6-29.5)	6.9	6.8
*Regular intake of healthy food (>= 5 days per week)*							
Fruits	32.7 (32.1-33.4)	32.6 (31.9-33.3)	2.0	1.9	33.3 (31.6-35.1)	2.7	3.0
Vegetables	37.7 (37.0-38.4)	36.8 (36.1-37.5)	3.1	2.5	42.8 (41.0-44.7)	4.8	4.5
Physical active (>=300min/week)	15.8 (15.4-16.2)	15.3 (14.9-15.8)	4.0	3.7	18.5 (17.4-19.6)	3.0	3.2
*Weight-related behavior*							
Use of laxative to lose weight	7.0 (6.7-7.3)	7.2 (6.9-7.6)	3.7	3.7	5.9 (5.2-6.6)	0.7	1.1
Use of medicine to lose weight	6.0 (5.7-6.3)	6.3 (5.9-6.6)	6.1	5.6	4.5 (3.9-5.2)	2.1	1.6
Use of medicine to gain weight	7.1 (6.7-7.4)	7.4 (7.1-7.9)	4.1	3.4	4.5 (3.9-5.2)	4.2	2.7

*: adjusted by gender, age, skin color/race and mother’s level of education

VPC: variance partition coefficients; IC_95%_: confidence interval 95%

There was significant between-school variance, mainly when the use of drugs was analyzed. In the private schools, almost 20% of the total variance of smoking and use of illegal drugs, and 8.5% of the alcohol consumption variance was between-school. The figures were very high in the public school as well, reaching 15.3% in the use of illegal drugs. Lower, but still significant, values were observed in the other outcomes. Particularly, the VPC of the regular intake of deep-fried salty snacks and soft drinks was 8.8% and 6.8% in the private schools and 3.7% and 5.1% in the public schools, respectively. The VPC of regular intake of healthy food and physical activity ranged between 1.9% and 4.5%. Finally, in the public schools 5.6% and 3.4% of the variance of using medicines to gain/lose weight was between-schools. In all outcomes, the VPC did not change significantly when adjusted by individual variables.

## DISCUSSION

The between-schools variance was significant in all tested models. The highest VPCs were observed when the use of legal and illegal drugs was analyzed. This result underscores the importance of the school context on adolescent health and highlights that school environment interventions may be effective for promoting healthy behaviors.

The school and its vicinity can influence student behavior in different ways. Schools located in poor or more violent neighborhoods may find it difficult to hire the most qualified teachers^3^ . Or the community may not have lobby power to demand better structure, staff or implementation of good-practices in education. The school can facilitate the engagement in healthy behavior by offering and stimulating opportunities to be healthy (e.g, availability of sports courts and physical education activities, offer healthy options in the cafeteria, etc)^4 , 5^ and by promoting barriers to unsafe/unhealthy behavior (e.g, not selling candies in the cafeteria, acting to prohibit retail sales of alcohol and tobacco in the vicinity, etc)^6^ . Also, the school must provide social support and improve student-staff relationships.

Health promotion in the school setting must be a public health priority. However, many actions have been based on ineffective intraclass educational activities^6^ . Public policies must focus on offering good physical structure, qualified and motivated staff, structured afterschool programs, good school climate and reduce the risks the adolescent are exposed to. At the school, the healthier choices must be the easier choices.

This study has limitations. First, the information was collected through self-report questionnaires. Depending on social norms, values and expectations, the adolescents may provide inaccurate answers on their health behaviors. The study, however, used smartphones to collect the data and the questionnaire was answered autonomously, increasing the chances of obtaining reliable information. Second, the study collected data from the students present in the school in the day that the questionnaire was applied. The absenteeism may be correlated to some variables analyzed.

The results of the present study reinforce that initiatives that improve the school context have potential to better adolescents’ health. It’s important that new researches explore which schools’ characteristics explain the contextual variation of each outcome. The cross-level interactions between individual, familial and contextual characteristics also need to be investigated deeper. Advancing the knowledge on the role of the schools’ context on the adolescents’ lives may lead to more effective and evidence-based strategies.
